# Does antiretroviral treatment change HIV-1 codon usage patterns in its genes: a preliminary bioinformatics study

**DOI:** 10.1186/s12981-016-0130-y

**Published:** 2017-01-07

**Authors:** Navaneethan Palanisamy, Nathan Osman, Frédéric Ohnona, Hong-Tao Xu, Bluma Brenner, Thibault Mesplède, Mark A. Wainberg

**Affiliations:** 1 McGill University AIDS Centre, Lady Davis Institute for Medical Research, Jewish General Hospital, 3755, Ch. Cote-Ste-Catherine, Montréal, QC Canada; 2 Department of Microbiology and Immunology, Faculty of Medicine, McGill University, Montréal, QC Canada; 3 The Hartmut Hoffmann-Berling International Graduate School of Molecular and Cellular Biology (HBIGS), University of Heidelberg, Heidelberg, Germany; 4 Molecular and Cellular Engineering Group, BioQuant, University of Heidelberg, Heidelberg, Germany

**Keywords:** HIV-1, Codon usage frequency, Bioinformatics, Antiretroviral therapy, Resistance

## Abstract

**Background:**

Codon usage bias has been described for various organisms and is thought to contribute to the regulation of numerous biological processes including viral infections. HIV-1 codon usage has been previously shown to be different from that of other viruses and man. It is evident that the antiretroviral drugs used to restrict HIV-1 replication also select for resistance variants. We wanted to test whether codon frequencies in HIV-1 sequences from treatment-experienced patients differ from those of treatment-naive individuals due to drug pressure affecting codon usage bias.

**Results:**

We developed a JavaScript to determine the codon frequencies of aligned nucleotide sequences. Irrespective of subtypes, using HIV-1 *pol* sequences from 532 treatment-naive and 52 treatment-experienced individuals, we found that *pol* sequences from treatment-experienced patients had significantly increased AGA (arginine; p = 0.0002***) and GGU (glycine; p = 0.0001***), and decreased AGG (arginine; p = 0.0001***) codon frequencies. The same pattern was not observed when subtypes B and C sequences were analyzed separately. Additionally, irrespective of subtypes, using HIV-1 *gag* sequences from 524 treatment-naive and 54 treatment-experienced individuals, gag sequences from treatment-experienced patients had significantly increased CUA (leucine; p < 0.0001***), CAG (glutamine; p = 0.0006***), AUC (isoleucine; p < 0.0001***) and UCU (serine; p = 0.0005***), and decreased AUA (isoleucine; p = 0.0003***) and CAA (glutamine; p = 0.0006***) codon frequencies.

**Conclusion:**

Using *pol* and *gag* genes derived from the same HIV-1 genome, we show that antiretroviral therapy changed certain HIV-1 codon frequencies in a subtype specific way.

## Background

HIV-1 can be classified into various groups (i.e. M, N, O and P). Viruses from groups M and N originated from independent transmissions of simian immunodeficiency virus (SIV) from chimpanzees to humans, while viruses from groups O and P originated from gorillas to humans [1]. Group M of HIV-1 is the most common worldwide and is further divided into various subtypes (i.e. A–K).

Since the identification of HIV as the etiological agent of AIDS more than 30 years ago, antiretroviral therapy has evolved to include the use of combinations of inhibitors that target two or more processes in HIV replication (e.g. entry, reverse transcription, DNA integration, maturation) to reduce viral replication [2, 3]. However, drug-resistant HIV mutants can often emerge during the course of therapy [4, 5]. Resistant viruses also exist among antiretroviral treatment-naive patients as a result of the transmission of drug resistant HIV variants [6].

Codon usage bias is defined as the preference for particular codon(s) over others in synthesis of the same amino acid. It is well known that codon usage bias exists among different organisms [7–9]. Codon usage bias might have arisen in the course of evolution to protect an organism from pathogens bearing invasive foreign nucleic acids, such as viruses and transposable elements, and is thus sometimes considered an aspect of intrinsic immunity. The importance of codon usage bias in the immune response is illustrated by the activity of the interferon inducible schlafen family member 11 (SLFN11) protein [10] that selectively inhibits late stages of HIV-1 production in a codon usage-dependent manner [10]. SLFN11 binds to tRNA and thereby prevents tRNA pool changes that would otherwise be triggered by HIV infection [10]. By using sequences documented over a period of 23 years, it has been shown that the codons of HIV regulatory genes match closely with human codon preference patterns, with *rev* being the closest followed *tat*, *nef* and *vpr* respectively [11]. It has been speculated that codon preference patterns that are similar to those of the host might confer several beneficial characteristics to HIV-1, including the potential for the emergence of drug resistance [11, 12].

Two hypotheses have been proposed to explain bias in codon usage. One of these involves the concept of translation efficiency, i.e. the genes of proteins that have to be expressed constitutively and/or in large quantities should have codon usage that is similar to that of the host cell, while the genes of proteins that have to be expressed under restrictive conditions and/or in small quantities might involve codon(s) that are not commonly used by the host cell. Re-engineering of the HIV-1 genome, such that its codons matched with the relative synonymous codon usage (RSCU) of humans, led to an increase in viral protein production [13].

The second hypothesis favours the notion that codon usage bias exists because of inherent genetic constraints (for e.g. GC contents) and associated mutation fixation probabilities, i.e. mutation biases [8]. These mutation fixation probabilities can be influenced by external factors such as the host immune system and antiretroviral drugs and this hypothesis is supported by a study that codons within parts of the HIV-1 *env* gene tend to match with human RSCU over the course of infection because of mutation pressure [14]. This led to the question whether antiretroviral therapy can change HIV codon frequencies significantly and ultimately the usage bias patterns. As a preliminary, to test our hypothesis, we have used HIV-1 *pol* and *gag* sequences from antiretroviral treatment-naive and treatment-experienced patients, retrieved from the Los Alamos HIV database, to see whether there are any significant difference in codon frequencies that may have resulted from treatment. As *env* sequences are highly variable and can be greatly influenced by the host immune system, we considered only *pol* and *gag* sequences in the present study. Due to limited information, we did not take into consideration additional clinical parameters that may have influenced our results such as regimen use and timing of treatment initiation, among others.

## Methods

**Fig. 1:**
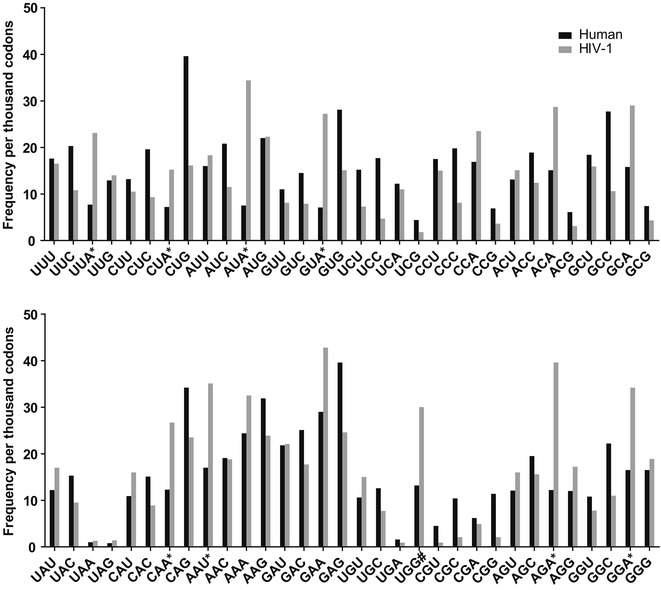
Comparison of codon usage between human and HIV-1. Codon usage data for human and HIV-1 were obtained from the codon usage database of Kazusa (http://www.kazusa.or.jp/codon/). Eight codons, i.e. UUA and CUA (leucine), AUA (isoleucine), GUA (valine), CAA (glutamine), AAU (asparagine), AGA (arginine) and GGA (glycine) were found to be present at levels > twofold in the HIV-1 genome compared to the human genome (represented by *). Tryptophan which has only one codon (i.e. UGG) is represented by #. An increase in UGG in HIV-1 simply means that tryptophan is more prevalent in HIV-1 than in human proteins

Codon usage data for man and HIV-1 were retrieved from the codon usage database of the Kazusa DNA Research Institute, Japan (http://www.kazusa.or.jp/codon/) (Fig. 1). Complete HIV-1 genome sequences (as nucleotides) from both antiretroviral treatment-naive and treatment-experienced patients were retrieved from the Los Alamos HIV database (http://www.hiv.lanl.gov/components/sequence/HIV/search/search.html) as multiple sequence aligned FASTA file. These genome sequences were collected and deposited at different time points from various geographical regions by others. We strictly took annotated sequences to make sure that the viral sequences used in this study were isolated from antiretroviral treatment-naive and treated-experienced patients. We additionally restricted the database to provide only one sequence per patient to eliminate bias. We chose *pol* and *gag* genes for this study because they are relatively conserved in HIV-1 compared to *env* gene [15]. Moreover, majority of HIV drugs currently available in the market are targeted to the *pol* region. From the complete genome, *pol* gene and *gag* gene sequences were cut out using BioEdit© software V.7.2.5 (http://www.mbio.ncsu.edu/bioedit/bioedit.html). The HIV-1 HXB2 *pol* and *gag* genes were used as a reference sequence. Using the same software, pol protein (and gag protein) multiple sequence alignments (by implementing ClustalW) were performed separately. Sequences with additional stop codons and poor sequence quality (including one or more R, Y and other nucleotides) were removed from further analysis. Nucleotides encoding amino acids from W34 to S53 in *pol* gene and amino acids at positions 1, 110–127, 371–374, 378, 385, 464–470, 475–484 and 497–499 in *gag* gene were also removed from further analysis because of difficulty with the alignment (i.e. this region was found to be highly prone to insertion-deletion mutations). We covered 98% of amino acids in *pol* gene and 91% of amino acids in *gag* gene in this study. Later, sequences were toggled back from amino acids to nucleotides. A java script was developed that gave us the codon usage per amino acid in Excel format (https://drive.google.com/folderview?id=0Bw4LWIJCCBxwRmVEalNNWG9JY1E&usp=sharing). The data were imported into GraphPad Prism V.5. We performed non-parametric test (Mann–Whitney test; 95% CI; two-tailed) for each codon between *pol* gene sequences derived from treatment-naive and treated individuals (same analysis repeated for *gag* gene sequences as well). We chose non-parametric tests over parametric tests for the entire study for two reasons: (1) we had fewer HIV-1 subtype C sequences from treatment-experienced patients, and (2) we did not have access to all relevant clinical parameters that would have assisted our statistical evaluations.

## Results

### Human and HIV-1 codon usage are different

Two earlier studies showed that HIV has different codon usage patterns compared to other viruses including HTLV-1 [16, 17], although, these previous works did not explain specific codon changes in detail. We compared codon usage in human and HIV-1 genomes (using data from Kazusa Codon Usage Database), and found that eight codons, i.e. UUA (leucine), CUA (leucine), AUA (isoleucine), GUA (valine), CAA (glutamine), AAU (asparagine), AGA (arginine) and GGA (glycine) were >twofold more common within the HIV-1 than in the human genome (Fig. 1, represented by *). UGG (tryptophan) was also overrepresented in HIV-1 compared to humans; however, given that UGG is the only codon for tryptophan, this observation simply indicates that this amino acid is more prevalent in HIV-1 than in human proteins (Fig. 1, represented by #). An earlier study also reported differences in codon usage patterns between HIV-1 and humans using HIV sequences obtained over 23 years [11].

### Phylogeny and resistance analysis of studied sequences

**Fig. 2:**
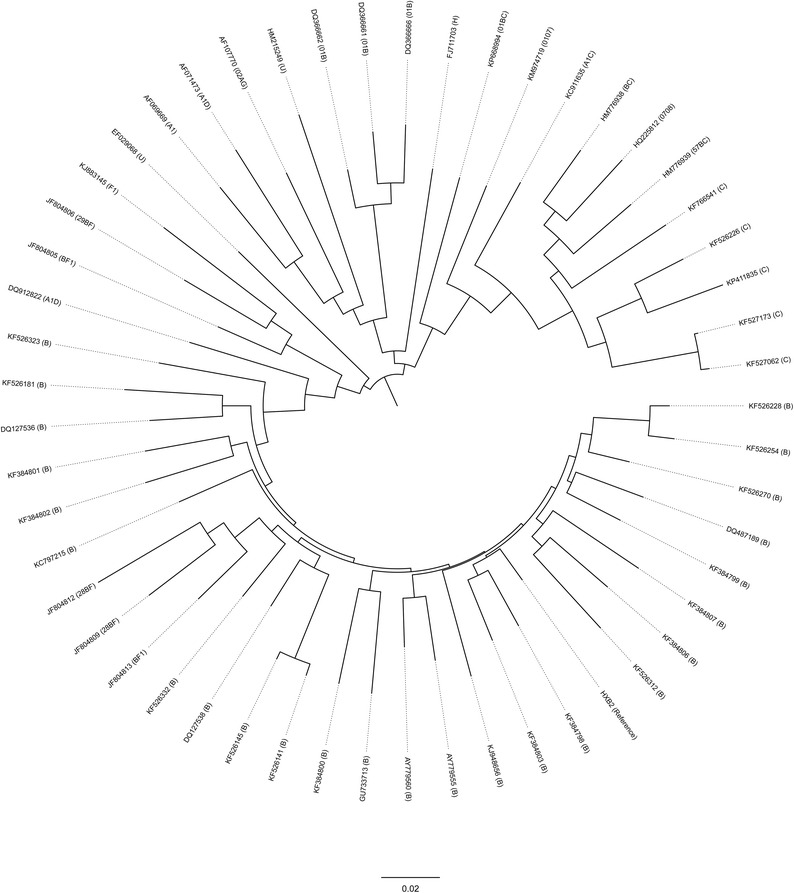
Phylogenetic analysis using HIV-1 *pol* gene sequences isolated from treatment-experienced patients. Maximum likelihood trees (Bootstrap method with 1000 replicates) were constructed using MEGA6 (http://www.megasoftware.net/) [18]. HIV-1 *pol* gene sequences used in this study were found to be diverse

First, we wanted to evaluate evolutionary relationships among the sequences used in this study. *pol* gene sequences from 532 treatment-naive and 52 treatment-experienced HIV-1 samples were studied. For the construction of a phylogenetic tree, MEGA6 (http://www.megasoftware.net/) software was used [18]. The tree construction parameters included: Maximum Likelihood (for statistical analysis), Bootstrap method (for testing of phylogeny), 1000 (for number of Bootstrap replications), nucleotides (for substitution type), Tamura-Nei model (for model) while others were set to default parameters. From the phylogenetic tree, we found that the sequences formed distinct diverse clusters, thereby making their sequences ideal for further analysis (Fig. 2).

**Fig. 3:**
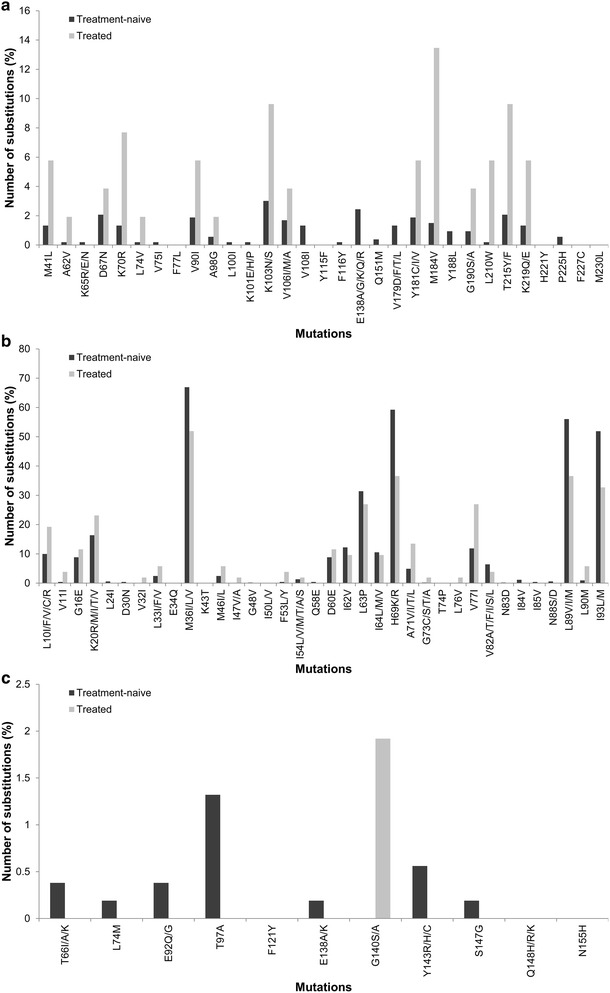
Percentages of resistance substitutions found in HIV-1 *pol* of treatment-naive and treatment-experienced individuals. **a** reverse transcriptase, **b** protease and **c** integrase

We also evaluated resistance mutations in treatment-naive and treated-experienced HIV-1 samples and included all the resistance markers within the *pol* gene, as listed by the International Antiviral Society—USA 2014 [19]. In the case of the reverse transcriptase (RT) gene, resistance markers were found to be more prevalent in HIV-1 samples isolated from treatment-experienced patients compared with treatment-naive patients but the same trend was not seen with resistance markers within the protease and integrase genes (Fig. 3). Two reasons for this might be a lower degree of protease and integrase resistance in treatment-experienced patients due to small sample size or because most patients had been prescribed RT inhibitors but not protease inhibitors or integrase inhibitors. For the RT region, mutations at amino acid positions 41, 70, 184, 190, 210 and 215 were found >fourfold more frequently in treatment-experienced than in treatment-naive patients.

### Certain HIV-1 codon frequencies in the pol gene are significantly different between treatment-naïve and -experienced patients

We investigated whether antiretroviral treatment influences HIV-1 codon frequency. Irrespective of HIV-1 subtype, we compared codon repartition within unique *pol* gene sequences of 532 treatment-naive and 52 treatment-experienced individuals with the following subtype distribution: B = 35.2, C = 38, AE = 9.4, others < 4% for treatment-naive and B = 53.9, BF = 9.6, C = 9.6, BC = 5.8 and others < 4% in treatment-experienced. Importantly, codon frequency was measured for each amino acid, thus excluding differences due to amino acid changes from this analysis. Of the eight above mentioned codons that were initially identified as being differentially used in humans vs HIV-1, one was significantly increased in sequences from treatment-experienced individuals, i.e. AGA (arginine) (p = 0.0002***) (Table 1). Additionally, GGU (glycine) was significantly increased (p = 0.0001***) in treatment-experienced compared to treatment-naive sequences. A different arginine codon, namely AGG, was significantly decreased (p = 0.0001***) in treatment-experienced sequences. Codons GCU and GCC of alanine, AAU and AAC of asparagine, GGG of glycine, CAU and CAC of histidine, AUU and AUC of isoleucine, CUG of leucine, CCG of proline and GUA of valine were also affected when we compared HIV-1 *pol* sequences from treatment-naive and treatment-experienced patients. While GCC, AAU, CAU, AUU and CUG were more prevalent in sequences from treatment-experienced individuals, GCU, AAC, GGG, CAC, AUC, CCG and GUA were decreased.

**Table 1:** Codon usage in *pol* and *gag* genes of HIV-1 in treatment-naive and treatment-experienced individuals

Amino acid	Codon	*pol*			*gag*		
		Naive (N = 532)	Treated (N = 52)	*P* value	Naive (N = 524)	Treated (N = 54)	*P* value
Alanine	GCU	15.60 ± 0.17	13.87 ± 0.52	0.0018**	23.10 ± 0.17	23.58 ± 0.65	0.7689
	GCC	19.66 ± 0.12	20.72 ± 0.42	0.0189*	18.38 ± 0.19	18.49 ± 0.58	0.9689
	GCA	63.25 ± 0.12	63.72 ± 0.34	0.1477	49.08 ± 0.22	48.46 ± 0.61	0.5766
	GCG	1.48 ± 0.07	1.68 ± 0.20	0.0932	9.43 ± 0.12	9.47 ± 0.40	0.7388
Arginine	CGU	0.09 ± 0.02	0.14 ± 0.08	0.3506	0.15 ± 0.03	0.20 ± 0.12	0.6272
	CGC	0.06 ± 0.02	0.05 ± 0.05	0.7593	0.23 ± 0.04	0.19 ± 0.11	0.8865
	CGA	3.33 ± 0.07	3.04 ± 0.15	0.2125	3.03 ± 0.10	3.30 ± 0.35	0.4781
	CGG	3.09 ± 0.05	3.41 ± 0.20	0.0621	5.58 ± 0.10	6.02 ± 0.33	0.2211
	AGA	60.65 ± 0.27	63.90 ± 0.72	0.0002***	56.80 ± 0.26	57.27 ± 0.78	0.5734
	AGG	32.77 ± 0.27	29.47 ± 0.71	0.0001***	34.20 ± 0.27	33.01 ± 0.90	0.0845
Asparagine	AAU	74.66 ± 0.25	76.56 ± 0.59	0.0189*	62.61 ± 0.34	63.48 ± 0.89	0.4616
	AAC	25.34 ± 0.25	23.44 ± 0.59	0.0189*	37.39 ± 0.34	36.52 ± 0.89	0.4616
Aspartic acid	GAU	59.40 ± 0.21	60.58 ± 0.59	0.1349	47.57 ± 0.40	49.08 ± 1.31	0.1854
	GAC	40.60 ± 0.21	39.42 ± 0.59	0.1349	52.43 ± 0.40	50.92 ± 1.31	0.1854
Cysteine	UGU	86.81 ± 0.33	86.76 ± 1.05	0.8285	80.66 ± 0.56	81.56 ± 1.45	0.8737
	UGC	13.19 ± 0.33	13.24 ± 1.05	0.8285	19.34 ± 0.56	18.44 ± 1.45	0.8737
Glutamic acid	GAA	72.48 ± 0.16	73.05 ± 0.62	0.2699	65.35 ± 0.27	66.64 ± 0.82	0.1509
	GAG	27.52 ± 0.16	26.95 ± 0.62	0.2699	34.65 ± 0.27	33.36 ± 0.82	0.1509
Glutamine	CAA	59.33 ± 0.15	59.62 ± 0.63	0.4549	59.93 ± 0.33	55.65 ± 1.05	0.0006***
	CAG	40.67 ± 0.15	40.38 ± 0.63	0.4549	40.07 ± 0.33	44.35 ± 1.05	0.0006***
Glycine	GGU	10.21 ± 0.08	11.24 ± 0.24	0.0001***	2.52 ± 0.11	3.16 ± 0.40	0.2888
	GGC	5.42 ± 0.10	5.45 ± 0.27	0.9708	21.22 ± 0.16	20.00 ± 0.52	0.0279*
	GGA	54.09 ± 0.15	54.47 ± 0.51	0.5641	46.95 ± 0.28	48.74 ± 0.72	0.0483*
	GGG	30.28 ± 0.16	28.84 ± 0.54	0.0076**	29.31 ± 0.23	28.10 ± 0.67	0.0936
Histidine	CAU	67.75 ± 0.35	70.96 ± 1.00	0.0043**	59.17 ± 0.56	63.59 ± 1.92	0.0136*
	CAC	32.25 ± 0.35	29.04 ± 1.00	0.0043**	40.83 ± 0.56	36.41 ± 1.92	0.0136*
Isoleucine	AUU	29.95 ± 0.11	30.74 ± 0.30	0.0073**	23.52 ± 0.25	22.33 ± 0.79	0.1297
	AUC	15.23 ± 0.09	14.46 ± 0.29	0.0325*	15.73 ± 0.25	19.56 ± 0.82	<0.0001***
	AUA	54.82 ± 0.08	54.80 ± 0.29	0.9037	60.75 ± 0.20	58.10 ± 0.81	0.0003***
Leucine	UUA	39.10 ± 0.14	39.49 ± 0.51	0.5880	44.21 ± 0.23	42.06 ± 0.79	0.0027**
	UUG	10.72 ± 0.11	10.75 ± 0.33	0.8305	13.01 ± 0.14	12.63 ± 0.43	0.6161
	CUU	12.19 ± 0.12	11.78 ± 0.32	0.1110	12.48 ± 0.15	12.01 ± 0.41	0.2454
	CUC	6.62 ± 0.06	6.30 ± 0.24	0.1421	10.57 ± 0.11	10.52 ± 0.38	0.9979
	CUA	19.65 ± 0.17	18.96 ± 0.48	0.2819	12.12 ± 0.25	15.19 ± 0.67	<0.0001***
	CUG	11.72 ± 0.11	12.71 ± 0.30	0.0054**	7.62 ± 0.13	7.59 ± 0.50	0.7665
Lysine	AAA	72.28 ± 0.14	72.47 ± 0.39	0.6021	66.80 ± 0.23	66.78 ± 0.72	0.9256
	AAG	27.72 ± 0.14	27.53 ± 0.39	0.6021	33.20 ± 0.23	33.22 ± 0.72	0.9256
Phenylalanine	UUU	65.69 ± 0.19	66.09 ± 0.71	0.5312	61.11 ± 0.36	58.32 ± 1.28	0.0214*
	UUC	34.31 ± 0.19	33.91 ± 0.71	0.5312	38.89 ± 0.36	41.68 ± 1.28	0.0214*
Proline	CCU	26.08 ± 0.13	26.53 ± 0.35	0.1652	29.76 ± 0.18	28.69 ± 0.61	0.1140
	CCC	18.13 ± 0.13	18.04 ± 0.40	0.8455	14.29 ± 0.17	13.69 ± 0.69	0.1897
	CCA	54.53 ± 0.10	54.64 ± 0.32	0.7969	52.49 ± 0.18	54.31 ± 0.73	0.0072**
	CCG	1.25 ± 0.07	0.80 ± 0.16	0.0330*	3.46 ± 0.13	3.32 ± 0.48	0.3902
Serine	UCU	6.62 ± 0.12	6.64 ± 0.33	0.9876	2.01 ± 0.11	3.38 ± 0.43	0.0005***
	UCC	3.41 ± 0.09	3.39 ± 0.31	0.7542	11.96 ± 0.14	12.59 ± 0.47	0.2148
	UCA	26.98 ± 0.14	26.83 ± 0.53	0.9924	32.96 ± 0.20	32.27 ± 0.73	0.7226
	UCG	0.77 ± 0.06	1.12 ± 0.21	0.0563	0.89 ± 0.09	1.28 ± 0.36	0.4649
	AGU	39.58 ± 0.23	39.35 ± 0.79	0.6742	15.17 ± 0.22	14.56 ± 0.71	0.3236
	AGC	22.63 ± 0.17	22.67 ± 0.52	0.7640	37.02 ± 0.24	35.91 ± 0.90	0.3998
Threonine	ACU	30.57 ± 0.12	30.39 ± 0.44	0.4910	25.24 ± 0.20	25.23 ± 0.62	0.8150
	ACC	13.88 ± 0.10	13.28 ± 0.36	0.1156	28.67 ± 0.20	28.53 ± 0.85	0.5475
	ACA	54.44 ± 0.11	55.11 ± 0.40	0.0659	44.77 ± 0.20	45.10 ± 0.73	0.8720
	ACG	1.11 ± 0.06	1.23 ± 0.19	0.6161	1.32 ± 0.10	1.14 ± 0.33	0.4743
Tyrosine	UAU	69.25 ± 0.17	68.61 ± 0.61	0.3502	86.34 ± 0.41	83.80 ± 1.47	0.2448
	UAC	30.75 ± 0.17	31.39 ± 0.61	0.3502	13.66 ± 0.41	16.20 ± 1.47	0.2448
Valine	GUU	15.04 ± 0.11	15.57 ± 0.34	0.1411	10.46 ± 0.20	11.03 ± 0.57	0.4377
	GUC	12.87 ± 0.10	12.85 ± 0.32	0.9003	9.38 ± 0.19	8.56 ± 0.62	0.1446
	GUA	58.66 ± 0.14	57.82 ± 0.37	0.0379*	57.59 ± 0.30	57.85 ± 0.99	0.5363
	GUG	13.43 ± 0.12	13.76 ± 0.36	0.2377	22.58 ± 0.25	22.57 ± 0.82	0.8879

Values are given as mean ± SEM in  %. p values <0.05*, <0.01**, and <0.001***

### HIV-1 codon frequency change is subtype specific

To try to determine a role for viral subtype, we analysed 187 HIV-1 subtype B sequences from treatment-naive individuals and compared them with 28 HIV-1 subtype B sequences from treatment-experienced individuals. None of the codons differed significantly (i.e. *** or ** significance). Only AUA (isoleucine) and GUC (valine) trended towards higher prevalence in treatment-experienced sequences and with low significance (i.e. p = 0.0443* and 0.0201* respectively).

We also compared 202 HIV-1 subtype C sequences from treatment-naive with 5 HIV-1 subtype C sequences from treatment-experienced individuals. Phylogenetic analysis (Fig. 2b) and sequence geography information showed that the 4 out of 5 HIV-1 subtype C sequences from treatment-experienced persons were evolutionarily distinct from one another. Serine codons i.e. UCC, AGU and AGC significantly differed in sequences from treatment-experienced individuals (i.e. p = 0.0052**, 0.0033** and 0.0085** respectively) with UCC and AGU found to be diminished while AGC was increased. UUA for leucine, GCU and GCA for alanine, and AGA and AGG codons for arginine all differed with p values of 0.0276* (for both lysine codons), 0.0423* (UUA), 0.038* (GCU), 0.0267* (GCA), 0.0138* (AGA) and 0.0397* (AGG). Codons GCA, AGA, and AAA were more frequent in sequences from treatment-experienced persons while the four other codons were less frequent in sequences from treatment-experienced individuals. No significant changes were seen in regard to other codons.

### Certain HIV-1 codon frequencies in the gag gene are significantly different between treatment-naïve and –experienced individuals

We also studied *gag* gene sequences from 524 treatment-naive and 54 treatment-experienced individuals (Table 1) with the following subtype distribution: B = 36.4, C = 38, AE = 9.9 and others < 4% in treatment-naive and B = 48.1, BF = 13, BC = 7.4, A = 5.6 and others < 2% in treatment-experienced persons. Of the eight differentially used codons (when compared between humans and HIV-1), CUA (leucine), AUA (isoleucine) and CAA (glutamine) were significantly changed (i.e. p < 0.0001***; increased, p = 0.0003***; decreased and p = 0.0006***; decreased respectively) when comparing treatment-naive with treatment-experienced HIV-1 sequences (Table 1). Additionally, CAG (glutamine), AUC (isoleucine) and UCU (serine) was found to be increased significantly (i.e. p = 0.0006***, p < 0.0001*** and p = 0.0005*** respectively) on treatment. Codons that only displayed minor changes in the aftermath of treatment were GGC and GGA (glycine), CAU and CAC (histidine), UUA (leucine), UUU and UUC (phenylalanine) and CCA (proline).

### The role of drug pressure, GC content and other factors?

The differences in codon frequency between treatment-naive and treatment-experienced sequences could conceivably be influenced by the emergence of resistance substitutions. Irrespective of subtype, an increase in AGA codon usage in *pol* could potentially be related to the prevalence of K70R substitutions associated with stavudine-based or zidovudine-based therapy (Fig. 3). Lysine (K) is encoded by two codons: AAA and AAG, the former of which can give rise to the AGA (arginine) codon through a single A to G transition. K70R substitutions in reverse transcriptase could therefore result in an increase in the proportion of AGA codons. Similar explanations can be proposed for treatment-associated changes in AAU codons (asparagine) that might be related to K103N substitutions (AAG or AAA to AAU) (Fig. 3). On the other hand, irrespective of subtype, there was a significant decrease (i.e. p = 0.0001***) in AGG (arginine) in treatment-experienced individuals. Although AAG (lysine) can undergo a single A to G transition to give rise to AGG (arginine), this situation is not favoured, indicating that amino acid substitutions due to drug pressure may not be alone sufficient to influence codon frequency patterns. When considering only the genomic region encoding for RT, we found that codons AGA (arginine) and AAU (asparagine) were significantly increased in sequences from treatment-experienced patients (i.e. p = 0.0250* and p = 0.0040**, respectively). Additionally, we found that codon GGU (glycine) was significantly more frequent in sequences from treatment-experienced individuals (i.e. p < 0.0001***). An increase in GGU codon might be attributed to the A98G substitution in RT.

## Discussion

Except tryptophan, each amino acid has more than one codon that can be decoded by the amino acid containing t-RNA. Codon usage bias is a measure of codon use for each amino acid and should not be reflected in baseline differences in peptidic sequences. Codon usage bias is likely important for the modulation of translation processes. Using *pol* and *gag* gene sequences from treatment-naive and treatment-experienced patients, we have shown that antiretroviral therapy can modulate codon frequencies that might ultimately lead to usage biases (Table 1). Although this is an initial attempt at this type of work, it was limited by the availability and diversity of numbers of sequences available from treatment-experienced patients.

A comparison of codon frequency differences between *pol* and *gag* in treatment-naive and treatment-experienced sequences showed that changes can occur at both the site of selection pressure, i.e. *pol,* and more distally i.e. *gag*. Whether these codon changes are due to functional constraints that potentiate mutations or to random events are not clear. Since the present study cannot be properly controlled, we recognize that additional cell culture and patient studies should be performed in order to generate relevant information about the processes of mutagenesis and codon frequency changes.

Of eight codons that were differentially expressed between HIV-1 and humans, AGA (arginine) in *pol* and CUA (leucine) in *gag* were significantly more prevalent (i.e. p = 0.0002*** and p < 0.0001***, respectively) in sequences from treatment-experienced persons while AUA (isoleucine) and CAA (glutamine), both in *gag*, were less frequent (i.e. p = 0.0003*** and p = 0.0006***, respectively) in treatment-experienced subjects. CAG (glutamine), AUC (isoleucine) and UCU (serine) in *gag* were also more prevalent (i.e. p = 0.0006***, p < 0.0001*** and p = 0.0005***, respectively) in treatment-experienced sequences.

Though the differences in codon frequencies of certain codons between treatment-naive and treatment-experienced sequences appear to be significant, it was not up to the level of changing the usage bias patterns indicating that it might be a slow or complex process. Further, one should also keep in mind that primary and secondary drug resistance mutations may affect codon frequencies, which makes this type of study further challenging. However, since treatment affects codon usage frequencies both in *pol* and *gag*, our results suggest that resistance mutations did not account for all changes in codon frequency. A limitation of this work that we will correct in future work is a paucity of sequences from treatment-experienced patients as well as relevant clinical information. In addition, we do not know if some of the patients who provided samples were members of a single cluster, which would limit diversity. Nonetheless, the concept of altered codon frequency and usage is important and could conceivably also apply to other viruses such as HCV or HBV.

## Conclusions

Using *pol* and *gag* genes derived from the same HIV-1 genome, we show that antiretroviral therapy changed certain HIV-1 codon frequencies in a subtype specific way. Future additional studies should be performed in order to generate relevant information about the processes of mutagenesis and codon frequency changes.

## Abbreviations

HIV-1: human immunodeficiency virus 1
AIDS: acquired immune deficiency syndrome
SIV: simian immunodeficiency virus
RSCU: relative synonymous codon usage
HTLV-1: human T-lymphotropic virus 1
HBV: hepatitis B virus
HCV: hepatitis C virus
SLFN11: Schlafen family member 11

## References

[1] D’Arc M, Ayouba A, Esteban A, Learn GH, Boue V, Liegeois F, Etienne L, Tagg N, Leendertz FH, Boesch C (2015). Origin of the HIV-1 group O epidemic in western lowland gorillas. Proc Natl Acad Sci USA.

[2] Arts EJ, Hazuda DJ (2012). HIV-1 antiretroviral drug therapy. Cold Spring Harb Perspect Med.

[3] Gunthard HF, Aberg JA, Eron JJ, Hoy JF, Telenti A, Benson CA, Burger DM, Cahn P, Gallant JE, Glesby MJ (2014). Antiretroviral treatment of adult HIV infection: 2014 recommendations of the international antiviral society-USA panel. JAMA.

[4] Iyidogan P, Anderson KS (2014). Current perspectives on HIV-1 antiretroviral drug resistance. Viruses.

[5] Jespersen S, Tolstrup M, Honge BL, Medina C, Te Dda S, Ellermann-Eriksen S, Ostergaard L, Wejse C, Laursen AL (2015). Bissau HIVcsg: high level of HIV-1 drug resistance among patients with HIV-1 and HIV-1/2 dual infections in Guinea-Bissau. Virol J.

[6] Su Y, Zhang F, Liu H, Smith MK, Zhu L, Wu J, Wang N (2014). The prevalence of HIV-1 drug resistance among antiretroviral treatment naive individuals in mainland China: a meta-analysis. PLoS ONE.

[7] Andersson SG, Kurland CG (1990). Codon preferences in free-living microorganisms. Microbiol Rev.

[8] Hershberg R, Petrov DA (2008). Selection on codon bias. Annu Rev Genet.

[9] Grantham R, Gautier C, Gouy M, Mercier R, Pave A (1980). Codon catalog usage and the genome hypothesis. Nucleic Acids Res.

[10] Li M, Kao E, Gao X, Sandig H, Limmer K, Pavon-Eternod M, Jones TE, Landry S, Pan T, Weitzman MD, David M (2012). Codon-usage-based inhibition of HIV protein synthesis by human schlafen 11. Nature.

[11] Pandit A, Sinha S (2011). Differential trends in the codon usage patterns in HIV-1 genes. PLoS ONE.

[12] Kijak GH, Currier JR, Tovanabutra S, Cox JH, Michael NL, Wegner SA, Birx DL, McCutchan FE (2004). Lost in translation: implications of HIV-1 codon usage for immune escape and drug resistance. AIDS Rev.

[13] Haas J, Park EC, Seed B (1996). Codon usage limitation in the expression of HIV-1 envelope glycoprotein. Curr Biol.

[14] Meintjes PL, Rodrigo AG (2005). Evolution of relative synonymous codon usage in human immunodeficiency virus type-1. J Bioinform Comput Biol.

[15] Coplan PM, Gupta SB, Dubey SA, Pitisuttithum P, Nikas A, Mbewe B, Vardas E, Schechter M, Kallas EG, Freed DC (2005). Cross-reactivity of anti-HIV-1 T cell immune responses among the major HIV-1 clades in HIV-1-positive individuals from 4 continents. J Infect Dis.

[16] Grantham P, Perrin P (1986). AIDS virus and HTLV-I differ in codon choices. Nature.

[17] Kypr J, Mrazek J (1987). Unusual codon usage of HIV. Nature.

[18] Tamura K, Stecher G, Peterson D, Filipski A, Kumar S (2013). MEGA6: molecular evolutionary genetics analysis version 6.0. Mol Biol Evol.

[19] Wensing AM, Calvez V, Gunthard HF, Johnson VA, Paredes R, Pillay D, Shafer RW, Richman DD (2014). 2014 update of the drug resistance mutations in HIV-1. Top Antivir Med.

